# Long Noncoding RNAs AC009014.3 and Newly Discovered XPLAID Differentiate Aggressive and Indolent Prostate Cancers

**DOI:** 10.1016/j.tranon.2018.04.002

**Published:** 2018-05-01

**Authors:** Anthony J. Cesnik, Bing Yang, Andrew Truong, Tyler Etheridge, Michele Spiniello, Maisie I. Steinbrink, Michael R. Shortreed, Brian L. Frey, David F. Jarrard, Lloyd M. Smith

**Affiliations:** ⁎ Department of Chemistry, University of Wisconsin-Madison, Madison, WI, USA; † Department of Urology, University of Wisconsin-Madison, Madison, WI, USA; ‡ Carbone Cancer Center, University of Wisconsin-Madison, Madison, WI, USA; § Genome Center of Wisconsin, University of Wisconsin-Madison, Madison, WI, USA

## Abstract

*INTRODUCTION:* The molecular mechanisms underlying aggressive versus indolent disease are not fully understood. Recent research has implicated a class of molecules known as long noncoding RNAs (lncRNAs) in tumorigenesis and progression of cancer. Our objective was to discover lncRNAs that differentiate aggressive and indolent prostate cancers. *METHODS:* We analyzed paired tumor and normal tissues from six aggressive Gleason score (GS) 8-10 and six indolent GS 6 prostate cancers. Extracted RNA was split for poly(A)+ and ribosomal RNA depletion library preparations, followed byRNA sequencing (RNA-Seq) using an Illumina HiSeq 2000. We developed an RNA-Seq data analysis pipeline to discover and quantify these molecules. Candidate lncRNAs were validated using RT-qPCR on 87 tumor tissue samples: 28 (GS 6), 28 (GS 3+4), 6 (GS 4+3), and 25 (GS 8-10). Statistical correlations between lncRNAs and clinicopathologic variables were tested using ANOVA. *RESULTS:* The 43 differentially expressed (DE) lncRNAs between aggressive and indolent prostate cancers included 12 annotated and 31 novel lncRNAs. The top six DE lncRNAs were selected based on large, consistent fold-changes in the RNA-Seq results. Three of these candidates passed RT-qPCR validation, including AC009014.3 (*P* < .001 in tumor tissue) and a newly discovered X-linked lncRNA named XPLAID (*P* = .049 in tumor tissue and *P* = .048 in normal tissue). XPLAID and AC009014.3 show promise as prognostic biomarkers. *CONCLUSIONS:* We discovered several dozen lncRNAs that distinguish aggressive and indolent prostate cancers, of which four were validated using RT-qPCR. The investigation into their biology is ongoing.

## Introduction

There is currently no standard clinical assay to establish aggressive behavior in prostate cancer, although this is an active area of interest [1]. The standard method for evaluating prostate cancer prognosis involves visual evaluation of prostate tissue biopsies. Each biopsy is assigned a Gleason score [2], which combined with clinical stage provides some prognostic information. This score is predictive of survival [3], but it requires an invasive biopsy. Differentiation of aggressive and indolent prostate cancer subtypes with a molecular clinical assay would enable better informed decision making as to the course of treatment.

The problem of delineating the molecular differences contributing to tumor aggressiveness has been notoriously difficult and the subject of numerous investigations. Several studies have examined prostate cancer cell lines or cohorts of tumor specimens to find genomic variants [4], DNA methylation patterns [5], RNA-binding proteins [6], gene expression [7], or protein expression patterns [8], [9] that are characteristic of aggressive or indolent cancers. Another promising avenue towards an understanding of the differences between these cancers is identifying and characterizing long noncoding RNAs (lncRNAs). These RNA molecules (defined to be longer than 200 bp and not translated into proteins) have diverse cellular functions [10] and are known to be associated with subtypes of prostate cancer [11]. Certain lncRNAs implicated in prostate cancer have been thoroughly investigated. One such RNA, SChLAP1, is significantly overexpressed in aggressive prostate cancers and shows promise as a prognostic indicator; this molecule acts to promote metastasis by binding to a tumor-suppressing complex, inhibiting its beneficial properties [12]. Another lncRNA named PCA3 is found at higher levels in prostate cancer [13] but has limited ability to distinguish grade. These limited examples demonstrate a role for lncRNAs and their potential as biomarkers.

In this study, we developed a workflow for global analysis of lncRNAs using deep RNA sequencing (RNA-Seq) data. The discovery of lncRNAs requires tools for aligning RNA-Seq reads, reconstructing full-length transcripts from read alignments, and annotating noncoding transcripts. For each of these purposes, we chose to use software (*STAR* [14], *cufflinks* [15], [16], and *slncky* [17], as detailed in the Methods) that demonstrated excellent consistency for alignments and capabilities for annotation. The combination of these tools adds an effective workflow to the growing set of tools for lncRNA discovery [18], [19], [20]. We used this strategy to analyze RNA-Seq data collected for prostate tissue specimens to find new targets that show potential as prognostic indicators of prostate cancer aggressiveness.

## Materials and Methods

**Figure 1:**
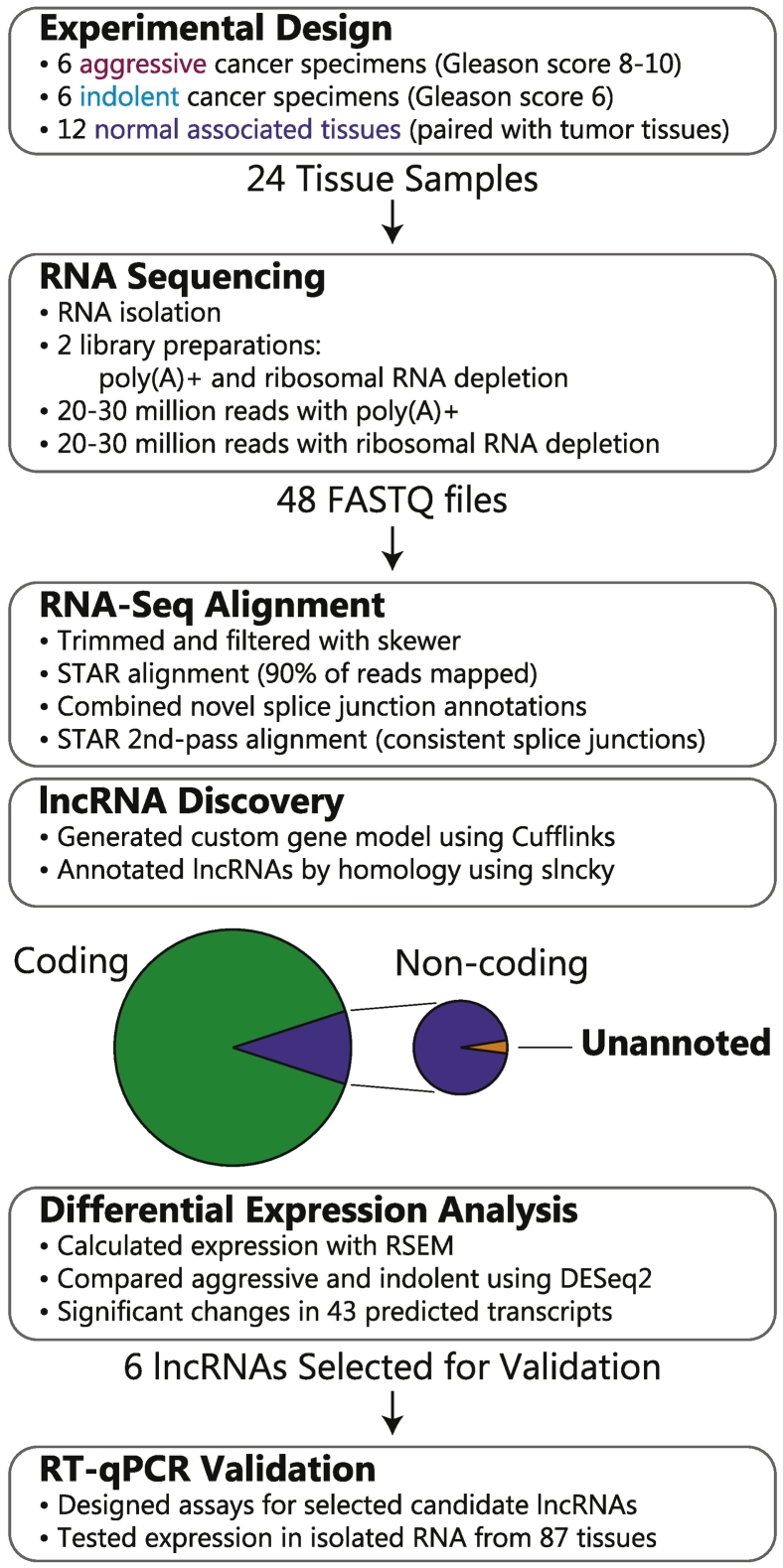
This study takes advantage of recent advances in RNA-seq analysis to discover lncRNAs present in primary tissue samples. These lncRNAs are evaluated to find candidates that are then validated using RT-qPCR.

This study focuses on identifying novel lncRNAs that differentiate aggressive and indolent prostate cancers using RNA sequencing data collected from primary tissue samples. An overview of the approach is presented in Figure 1.

### Primary Tissue Specimens

Twelve radical prostatectomy specimens were obtained from the University of Wisconsin Comprehensive Cancer Center (UWCCC) BioBank, six from patients with high-grade cancer and six from patients with low-grade cancer; both tumor and normal tissues were obtained from these specimens. Use of these human specimens was approved by Institutional Review Boards at the University of Wisconsin-Madison. A hematoxylin and eosin (H&E) slide was provided with the tissue blocks and marked by a genitourinary pathologist, indicating where the normal and tumor tissues were located. Then, areas containing >80% tumor and distant normal associated tissue were cored using the matching H&E slide. The selected cancers consist of six with Gleason scores of 8-10 (high grade, aggressive) and six with Gleason scores of 6 (low grade, indolent). With tumor and normal tissues isolated for each specimen, a total of 24 samples were analyzed (12 tumor, 12 normal). Tissues from each patient were minced on dry ice and mixed well to ensure sample uniformity before analysis by RNA-Seq.

### RNA Sequencing

Portions of each tissue were used for RNA extraction (see Supplemental Information) and subsequent RNA-Seq analysis. Each sample of 2 *μ*g of RNA was split in half for a) polyadenosine (poly(A)+) capture and b) ribosome-RNA depletion (rRNAd). Both groups were hybridized to an Illumina-HiSeq 2000 plate. RNA-Seq analysis was performed on each of these 48 samples, poly(A)+ and rRNAd for each of the 24 tissues. These data were analyzed to identify and quantify novel lncRNA molecules. For each of these 48 experiments, we collected 20 to 30 million paired-end reads on the Illumina HiSeq 2000 platform (Table S1). These reads were unstranded and 101 bp in length. (We note in hindsight that acquiring stranded reads would be beneficial, allowing evaluation of antisense lncRNAs, which have important biological functions [21]).

### RNA-Seq Analysis

#### RNA-Seq Alignment

Before aligning reads to the human genome, *skewer* [22] was used to trim adapter sequences and filter the average quality of reads to *Q* = 19 (version 0.1.127). Counts of reads after trimming and filtering are shown in Table S1. Then, RNA-Seq reads from both poly(A)+ and rRNAd library preparations were aligned to the human genome. To do this, we used the two-pass alignment protocol with *STAR* [14] (version 2.5.0b); the first-pass finds the superset of all novel splice junctions that are then used in the second-pass search to improve the consistency of alignment and quantification across these spliced transcripts.

*STAR* requires specially constructed indices based on the human genome to perform fast alignments of RNA-Seq reads. These indices were built using a human genome reference from Ensembl (the chromosomes and scaffolds from GRCh38.81 listed in Table S2 were used). Binary alignment map (BAM) files were sorted automatically with the “–outSAMtype BAM SortedByCoordinate” option, and noncanonical splice junctions were filtered using the “–outFilterIntronMotifs RemoveNoncanonical” option. Most of the trimmed reads were successfully mapped to the reference genome, as indicated by the high (>90%) mapping percentages in Table S1.

#### Transcript Reconstruction

To allow for the discovery of novel transcript isoforms, we used genome-guided transcript reconstruction software *Cufflinks* [15], [16] (version 2.2.1). This software uses the alignments of the short RNA-Seq reads to predict known and novel transcript isoforms. These predictions include large (>10 kb) regions that are not annotated as being transcribed in the reference gene model, possible novel lncRNAs, as well as small extensions of annotated transcripts. Because the poly-(A)+ and rRNAd libraries contain different types of RNAs, we performed this step separately for these two types of data. We note that reconstructing full-length, putative transcripts from short read fragments is difficult.

#### lncRNA Prediction

Transcript reconstruction with *Cufflinks* leads to a plethora of predicted transcript isoforms, around double the number of transcripts annotated in the reference gene model. To focus on likely noncoding transcripts, we used *slncky* teChen (version 1.0) to filter out predicted transcripts that share homology with coding transcripts in the human and mouse genomes. The remaining transcripts (thousands of them) are putative lncRNAs, and several hundred had no nearby annotations.

Several steps were required to use *slncky* on *Cufflinks* results. First, the transcripts predicted for each tissue sample were combined by running a related tool, *Cuffmerge*, on transcript models output by *Cufflinks*. This resulted in two combined transcript models, one for poly-(A)+ data and another for rRNAd data. Then, the resulting GTF files were converted to BED files containing UCSC annotations (hg38) that could be used by *slncky*.

#### Differential Expression

The expression of the predicted transcript isoforms was evaluated using *RSEM* [23] (version 1.2.25), which calculates transcript abundances based on RNA-Seq alignments. First, a reference transcriptome (consisting of all transcripts annotated in the reference gene model) was constructed using *RSEM*, which also directed the construction of *STAR* [14] (version 2.5.0b) indices for a subsequent alignment of trimmed RNA-Seq reads to these transcript sequences.

The relationship of tumor grade and the transcript expression patterns was evaluated using the software *DESeq2* [24] (version 1.12.3), which performs differential expression analysis. Transcript expression data for each sample were input as the expected read counts that were output for each transcript by *RSEM*. These counts were first normalized with respect to the library size of each sample using the regularized logarithmic transformation available within *DESeq2*. Then, the transcript expression in high-grade tumors was compared to the expression in low-grade tumors to find transcripts that exhibited significant differences between these cancers (*P* value <.01, corrected for multiple testing using the Benjimini-Hochberg correction [25]). The same analysis was used to find transcripts that distinguished normal associated tissues between patients with high- and low-grade cancers.

In conclusion, this RNA-Seq analysis workflow allows the discovery of lncRNAs and evaluation of their differential expression between two conditions. We are continuing to develop this workflow, including making it more user friendly by incorporating it into a new RNA-Seq analysis tool named Spritz (https://smith-chem-wisc.github.io/Spritz/); we note that this tool is in development and was not used in the present work.

### Validation of lncRNA Candidates in Primary Tissue RNA Samples Using RT-qPCR

When preparing RNA samples from primary tissue samples for RNA-seq, we set aside RNA samples for validation using qPCR and prepared them on ice as follows. Samples of RNA from previous experiments [26] were also included, adding intermediate-grade cancers (Gleason score of 7) to the sample set for validation; this set of 87 tissue samples was comprised of 56 indolent tumor samples (Gleason score 6 and 7 (3+4)) and 31 aggressive tumor samples (Gleason score 7 (4+3), 8, 9, and 10). Each of these RNA samples was reverse transcribed to cDNA for qPCR analysis using the qScript cDNA Supermix (Quantabio). Then, 2 *μ*l of cDNA sample was combined with 0.5 *μ*l of both forward and reverse primers (Table S3), 7 *μ*l of water, and 10 *μ*l of SYBR green mix from the PerfeCTa SYBR Green SuperMix kit (Quantabio). These samples were prepared in clear Multiplate 96-well PCR plates (BioRad), mixed using a microchannel pipette, and then spun down at 1000 RPM for ∼1 minute to eliminate bubbles in the mixtures. RT-qPCR was performed using a CFX96 RealTime PCR instrument (BioRad). Negative controls, using water instead of cDNA, were used to ensure that no contamination was introduced prior to RT-qPCR.

## Results

### Discovery of Novel lncRNAs in Aggressive and Indolent Prostate Cancers

**Figure 2:**
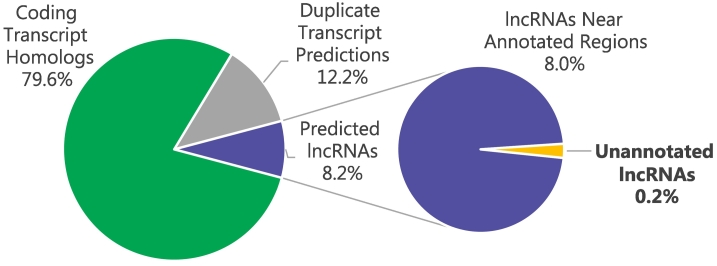
Reconstructed transcripts were compared to known coding transcripts in human and mouse showing that around 8% of transcripts in the model shared no homology and were likely noncoding. This class of molecules, i.e., lncRNAs, is of particular interest because they are relatively uncharacterized and known to be associated with prostate cancer [11]. A small portion of these putative lncRNAs was particularly interesting in that they were near no other annotations in the reference gene model, thus representing novel transcripts. These results allowed us to prioritize for further investigation several lncRNAs that differentiated aggressive and indolent cancers.

To discover novel lncRNAs, we reconstructed transcripts from RNA-Seq read alignments. These models contained 368,280 and 355,306 putative transcripts for poly-(A)+ and rRNAd libraries, respectively, that we used to quantify predicted lncRNAs. These counts are significantly larger than the 198,634 transcripts present in the Ensembl gene model reference because there are regions with many overlapping transcript predictions. To find likely lncRNAs amongst these many putative transcripts, we used the recently developed program *slncky* [17] to test whether transcripts shared homology with coding transcripts. As shown in Figure 2, coding homologs represented 79.6% of all predicted transcripts (338,596 for the poly-(A)+ model and 317,190 for the rRNAd model), and 8.2% were predicted to be lncRNAs (29,684 for the poly-(A)+ model and 38,116 for the rRNAd model). The remaining 12.2% were duplicate transcript predictions of either coding homologs or lncRNAs. Of the 30,000 to 40,000 predicted lncRNAs, several hundreds to just over one thousand were unannotated (novel) transcripts (664 for the poly-(A)+ model and 1173 for the rRNAd model). By connecting these annotations to the results of differential expression analysis, we were able to find novel lncRNAs that differ between aggressive and indolent prostate cancers.

The abundances of the putative transcripts were calculated for each of the 48 tissue samples based on RNA-Seq alignments, and they were then analyzed for differential expression between aggressive and indolent cancers. Several hundred transcripts, including some lncRNAs, exhibited significant differences (Table 1). A total of 43 lncRNA transcripts passed manual inspection, during which alignment maps were inspected using the computer program *IGV* [27], [28], which can visualize read densities along regions of the genome, to verify that clear, marked differences in read count were observed across each region called significant. These results agree with other reports; specifically, we found 662 DE transcripts by comparing tumors from indolent (Gleason 6) and aggressive (Gleason ≥ 8) cancers using rRNAd library preparation, and Presner and colleagues [12] found 559 DE transcripts by the same comparison using the same library preparation method.

**Table 1:** RNA-Seq Data Analysis Revealed Dozens of lncRNAs that Exhibited Differential Expression Between Aggressive and Indolent Cancers

Tissue	Library Preparation	# DE Transcripts	# DE lncRNA
T	poly-(A)+	1438	88
N	poly-(A)+	1051	65
T	rRNAd	662	87
N	rRNAd	534	47

The 43 differentially expressed (DE) lncRNAs between aggressive and indolent cancers were comprised of 12 annotated lncRNAs and 31 novel lncRNAs (Table S4). The annotated ones include SChLAP1, an lncRNA known to be associated with aggressive prostate cancers [12]. In the same gene desert as SChLAP1, we found 20 novel DE lncRNAs and 1 other annotated transcript. Other notable DE lncRNAs include three in a gene desert of the X-chromosome (TCONS_00394362, TCONS_00400757, and TCONS_00400766) and a novel lncRNA (TCONS_00201747) that was differentially expressed in both tumor and in normal associated tissue. Interestingly, several of these lncRNAs demonstrated differential expression in nontumor tissue from patients with aggressive and indolent cancer, suggesting a field defect in tumor generation. AC009014.3 was found to be overexpressed in indolent tissues compared to aggressive cancers.

### XPLAID and AC009014.3 Are lncRNAs That Exhibit Potential as Prognostic Indicators of Prostate Cancer Aggressiveness

Of the 43 differentially expressed lncRNAs (Table S4), 6 were further validated with RT-qPCR (Table 2). Each candidate was selected because it exhibited a fold-change that was large (>8-fold) and consistent (having at least three samples with abundances, *x*, that satisfied either $x_{\mathrm{aggressive}}>{\left(\bar{x}+3\sigma \right)}_{\mathrm{indolent}}$ or $x_{\mathrm{indolent}}>{\left(\bar{x}+3\sigma \right)}_{\mathrm{aggressive}}$). Filtering by these criteria gave 31 candidates. To narrow in on a smaller subset of candidates, we first selected lncRNAs with high abundances (C1, C2, C6). We then prioritized candidates with unique locations or behaviors: candidate C2 overlaps SChLAP1, a known lncRNA indicator of aggressive prostate cancers [12]; C3 and C4 are located on the X chromosome, evoking the well-known link between X-chromosome variations and prostate cancer [29]; C5 was the only candidate to show significantly elevated expression in indolent cancers; and C6 appears to be an extension of SChLAP1.

**Table 2:** Long Noncoding RNA Candidates That Were Selected for Further Validation

Candidate^a^	lncRNA Type	Tissue	Chrom^b^	Transcript Abundance^c^
C1	Novel	T	19	9.905, 0.425
		N		10.358, 0.688
C2 – SChLAP1	Annotated	T	2	5.510, 0.145
C3 – XPLAID	Novel	N	X	1.745, 0.058
C4	Novel	N	X	1.538, 0.032
C5 – AC009014.3	Annotated	T	5	0.0328, 3.458
C6	Novel	T	2	9.013, 0.295

a Transcript IDs: C1: TCONS_00201747, C2: TCONS_00220343, C3: TCONS_00394362, C4: TCONS_00400757, C5: TCONS_00320203, C6: TCONS_00235780.

b For more information on the genomic coordinates of these transcripts, see Table S5.

c Average transcript abundances for prostate cancer subtypes (aggressive, indolent) are presented in units of transcripts per million, TPM.

RT-qPCR results for the lncRNAs C5 and C6 were the most significant in distinguishing aggressive and indolent disease (Table 3; these results were tested using two-tailed *t* tests with equal variances, and *p* < .05 was the threshold for significance). In addition, candidates C2 and C3 had power to discriminate high- and low-grade cancers in tumor tissues and in normal associated tissues. Candidate C3 was accordingly named XPLAID, i.e., X-linked prostate lncRNA for adenocarcinoma indolence discrimination. Both the test of SChLAP1 (candidate C2) and the novel extended region of this transcript (candidate C6) showed elevated expression in highly aggressive tumor samples. Candidates C1 and C4 were not validated with this RT-qPCR dataset.

**Table 3:** Differences in Expression Exhibited by Selected RNAs between Low- and High-Grade Cancers

Candidate^a^	Grade^b^	Tumor Tissue		Normal Tissue	
		ΔC_T_ Mean (± SD)^c^	*P* Value^d^	ΔC_T_ Mean (± SD)^c^	*P* Value^d^
C1	Low (*n* = 40)	7.00 ± 2.01	.381	8.14 ± 2.08	.649
	High (*n* = 27)	6.55 ± 2.04		7.91 ± 2.27	
C2 – SChLAP1	Low (*n* = 38)	7.37 ± 2.36	**.036**	8.34 ± 2.12	**.017**
	High (*n* = 24)	5.60 ± 4.15		7.00 ± 1.71	
C3 – XPLAID	Low (*n* = 38)	12.23 ± 2.65	**.049**	12.97 ± 2.78	**.048**
	High (*n* = 24)	10.71 ± 3.26		11.41 ± 2.75	
C4	Low (*n* = 35)	12.25 ± 2.59	.229	13.05 ± 2.86	.266
	High (*n* = 24)	11.33 ± 3.26		12.10 ± 3.07	
C5 – AC009014.3	Low (*n* = 31)	8.23 ± 2.20	**.0002**	9.06 ± 2.02	.505
	High (*n* = 22)	10.65 ± 2.18		9.42 ± 1.78	
C6	Low (*n* = 41)	5.68 ± 2.03	**.015**	6.41 ± 1.91	.809
	High (*n* = 27)	4.42 ± 2.03		6.30 ± 1.99	

a Transcript IDs: C1: TCONS_00201747, C2: TCONS_00220343, C3: TCONS_00394362, C4: TCONS_00400757, C5: TCONS_00320203, C6: TCONS_00235780.

b Low grades are Gleason 6 & 7 (3+4); high grades are Gleason 7 (4+3), 8, 9, & 10. Sample number varied due to sample availability.

c Lower RT-qPCR thresholds (ΔC_T_) represent higher target expression.

d Using Welch’s *t* test with equal variances.

Although our aim was to differentiate high- and low-grade cancer, we note that candidates C1, C2, and C6 showed higher expression in tumors compared to normal associated tissues (Table S6). When separating these results by tumor grade (Table S7), C1 also differentiated tumor and normal associated tissues for both high- and low-grade cancers.

Finally, we evaluated the association of lncRNA expression with multiple clinicopathologic variables (Table 4; each association was tested using ANOVA with equal variances, and *P* < .05 was the threshold for significance). This analysis showed C2 is associated with higher tumor volume and PSA failure (this failure consists of showing a PSA concentration greater than 0.2 ng/ml after local therapy, indicating possible local recurrence following prostatectomy or radiation). C3 is associated with higher tumor volume. Interestingly, lower expression of C5 is related to higher tumor stage, higher tumor volume, and extracapsular extension.

**Table 4:** Differences in Expression Exhibited by SChLAP1 (C2), XPLAID (C3), and AC009014.3 (C5) in Prostate Tumor Tissues and Their Associations with Clinicopathologic Variables Assigned to Those Tumors

Clinicopathologic Variable	C2 ΔC_T_ Mean (± SD)^a^	*n*	*P* Value^b^	C3 ΔC_T_ Mean (± SD)^a^	*n*	*P* Value^b^	C5 ΔC_T_ Mean (± SD)^a^	*n*	*P* Value^b^
Tumor Stage									
T2a-T2c	7.28 ± 3.08	32	.774	11.27 ± 3.09	32	.135	8.80 ± 2.32	31	**.029**
T3a-T3b	6.97 ± 3.21	12		12.78 ± 2.42	12		10.58 ± 2.27	12	

Tumor Volume									
<10%	7.52 ± 2.58	13	**.011**	13.17 ± 2.83	14	**.041**	7.34 ± 1.73	10	**.021**
10%-30%	7.40 ± 3.13	31		10.79 ± 2.91	30		9.55 ± 2.30	30	
≥30%	4.70 ± 3.32	17		11.86 ± 2.80	17		10.00 ± 2.83	12	

PSA Failure^c^									
Yes	5.13 ± 2.78	17	**.0041**	11.12 ± 2.54	16	.351	10.10 ± 2.43	15	.075
No	7.56 ± 2.84	42		11.93 ± 3.05	43		8.77 ± 2.37	37	

Extracapsular Extension									
Yes	6.10 ± 3.35	16	.240	11.92 ± 2.69	17	.731	10.39 ± 2.08	14	**.025**
No	7.14 ± 2.87	43		11.63 ± 3.03	42		8.70 ± 2.43	38	

Positive Lymph Nodes									
Yes	3.62 ± 1.24	3	.056	11.54 ± 3.69	3	.918	10.80 ± 0.07	2	.335
No	7.03 ± 2.99	56		11.72 ± 2.91	56		9.09 ± 2.46	50	

Metastasis									
Yes	3.62 ± 1.24	3	.056	11.54 ± 3.69	3	.918	10.80 ± 0.07	2	.335
No	7.03 ± 2.99	56		11.72 ± 2.91	56		9.09 ± 2.46	50	

a Lower RT-qPCR thresholds (ΔC_T_) represent higher target expression.

b Using ANOVA tests with equal variances.

c PSA failure defined as PSA > 0.2 ng/mL.

## Discussion

We report three lncRNAs that differentiate aggressive and indolent prostate cancers: the well-known SChLAP1 transcript, AC009014.3, and a newly discovered transcript named XPLAID that is transcribed from a gene desert on the X-chromosome, a location that evokes the linkage [29] between X-chromosome variations and hereditary prostate cancer. These molecules show promise as prognostic biomarkers; SChLAP1 and XPLAID have elevated expression in both tumor and normal associated tissues of aggressive cancers, and AC009014.3 has elevated expression in tumors of indolent cancers. The elevated expression of AC009014.3 in indolent cancers points towards the possible utility of this lncRNA as an inhibitor of cancer progression.

Accessing tumor tissue requires invasive biopsies, and so analyzing normal associated tissues in addition to tumors offers valuable insight into the potential clinical uses of new lncRNA targets as analytes in noninvasive or minimally invasive prognostic assays. Previous studies have shown that prostate cells may circulate in the bloodstream [30] and are present in urine following aggressive prostate massage [31]; it is possible to analyze biomarkers in circulating tumor cells [32], prostate cells in urine [33], [34], and prostate exosomes in urine [35]. Specifically, SChLAP1 has previously been shown to be an effective biomarker of aggressive cancers in such urine analysis [36]. In our results, SChLAP1 and XPLAID exhibited significant differences of expression in normal tissues associated with aggressive cancer. We found no other report that SChLAP1 has elevated expression specifically in normal tissues associated with high-grade cancer; this expression difference in tissue that is readily available to urine analysis likely contributed to its success as a biomarker for that analysis. While XPLAID is expressed at lower abundance than SChLAP1, the significant difference of expression in normal tissues indicates XPLAID may also be amenable to this type of noninvasive assay. Future functional analysis of AC009014.3 and XPLAID may shed light on potential roles these transcripts play in promoting indolent and aggressive prostate cancers.

## Author Contributions

A. J. C. developed and executed the pipeline for discovering lncRNAs. A. T. and B. Y. validated the lncRNA candidates in primary tissue samples using RT-qPCR. B. Y. acquired the primary tissue samples from the UWCCC BioBank and prepared samples for RNA-Seq. T. E. performed a chart review of the twelve patients and their outcomes. M. S., M. I. S., and B. Y. designed and tested the RT-qPCR assays. M. S. did literature searches on the 43 differentially expressed lncRNAs; A. J. C. chose the six candidates based on this information. A. J. C., B. Y., M. R. S., B. L. F., D. F. J., and L. M. S. designed the study. D. F. J. and L. M. S. provided oversight of the entire study. A. J. C. drafted the manuscript, and all authors reviewed and made final edits on the manuscript.

## Competing Financial Interests

The authors declare no competing financial interests.

## Appendix A Supplementary data

Supplementary material Image 1
