# Supplementary material for: Long Noncoding RNAs AC009014.3 and Newly Discovered XPLAID Differentiate Aggressive and Indolent Prostate Cancers
Source: Transl Oncol. 2018 May 1;11(3):808–14. doi: 10.1016/j.tranon.2018.04.002 (PMC6154865; doi:10.1016/j.tranon.2018.04.002)
Supplement: Supplementary file 1 — Supplementary material [file mmc1.pdf]

Long Noncoding RNAs AC009014.3 and Newly Discovered XPLAID Differentiate Aggressive and Indolent Prostate Cancers

Supplementary Information

Anthony J. Cesnik[1], Bing Yang[2,3], Andrew Truong[2,3], Tyler Etheridge[2,3], Michele Spiniello[1], Maisie I. Steinbrink[1], Michael R. Shortreed[1], Brian L. Frey[1], David F. Jarrard[2,3], and Lloyd M. Smith[1,4,*]

[1]Department of Chemistry, University of Wisconsin-Madison, Madison, WI, USA
[2]Department of Urology, University of Wisconsin-Madison, Madison, WI, USA
[3]Carbone Cancer Center, University of Wisconsin-Madison, Madison, WI, USA
[4]Genome Center of Wisconsin, University of Wisconsin-Madison, Madison, WI, USA
[*]Corresponding author. Tel: +1 608 263 2594. E-mail: smith@chem.wisc.edu.

March 30, 2018

Contents

1 RNA Sequencing Methods **S-3**

List of Tables

S1 RNA-Seq experiments and alignment results for each sample and for the two library preparation methods. S-4
S2 Chromosomes and scaffolds from the GRCh38.81 primary assembly that were used for RNA-Seq analysis. S-5
S3 Primers used for RT-qPCR validation of the six lncRNA candidates. S-6
S4 Transcripts that were found to be significantly different by RNA-Seq differential expression analysis. S-7
S5 Coordinates and cufflinks attributes for each reconstructed transcript that exhibited significant differences between aggressive and indolent cancers. S-11
S6 Differences in expression exhibited by selected RNAs between normal and tumor prostate tissues. S-15
S7 Differences in expression exhibited by selected RNAs between normal and tumor prostate tissues, graded by Gleason score. S-16

1 RNA Sequencing Methods

Portions of each tissue were used for RNA-Seq analysis. The RNA was extracted using a PerfectPure RNA Tissue Kit (Thermo Fisher Scientific, Fitchburg, WI), homogenized in RNA lysis buffer, bound to an RNA affinity column, and digested with DNase to remove any DNA contamination, and then washed and eluted with nuclease-free water. RNA was quantified with a Nanodrop 1000 (Thermo Fisher Scientific, Fitchburg, WI), which determines concentration and purity of the RNA. The concentration required for RNA-Seq is > 50 ng/μl. The purity of RNA was measured by the Nanodrop, as determined by both the ratio of absorbances for nucleotides and proteins (A_{260nm}/A_{280nm}) and by the ratio of nucleotide absorbance and that of salt, carbohydrates, and proteins (A_{260nm}/A_{230nm}). The integrity of the isolated RNA was tested with EukaryoteTotal RNA Pico assay and analyzed by an Agilent Bioanalyzer 2100 – Eukaryotic Total RNA Pico Series II (Agilent Technologies, Waldbronn, Germany). This method uses ribosomal RNA fragments (18S and 28S) to evaluate RNA integrity. Satisfactory RNA integrity is indicated by two clear single peaks for the 18S and 28S subunits, respectively, and an RNA integrity number (RIN) of > 6.5. Each sample of 2 μg was split in half for a) polyadenosine (poly(A)+) capture and b) ribosome-RNA depletion (rRNAd). Both groups were hybridized to an Illumina-HiSeq 2000 plate. RNA-Seq analysis was performed on each of these 48 samples, poly(A)+ and rRNAd for each of the 24 tissues. These data were analyzed to identify and quantify novel lncRNA molecules. For each of these 48 experiments, we collected 20-30 million paired-end reads on the Illumina HiSeq 2000 platform (Table S1). These reads were unstranded and 101 bp in length.

Table S1: RNA-Seq experiments and alignment results for each sample and for the two library preparation methods. Gleason scores of 6 indicate indolent cancers, and scores of 8 or 9 indicate aggressive cancers. All RNA-Seq reads were unstranded, paired-end reads and 101 bp in length before trimming.

**Patient**	**Tissue**	**Gleason Score**	**Filtered Read Count**		**% Aligned by STAR**	
			poly-(A)+	rRNAd	poly-(A)+	rRNAd
160	NAT	6	28,784,898	27,197,558	94.6%	97.4%
160	T	6	28,779,396	29,341,662	96.1%	98.0%
253	NAT	6	28,117,695	27,331,064	95.7%	97.9%
253	T	6	28,9123,52	26,674,505	97.2%	98.0%
557	NAT	6	29,423,236	23,402,529	97.8%	98.2%
557	T	6	29,855,186	22,647,543	97.2%	98.0%
693	NAT	6	26,751,621	27,603,170	96.5%	98.2%
693	T	6	32,303,869	27,122,891	96.3%	98.3%
954	NAT	6	24,977,125	25,280,887	97.5%	98.4%
954	T	6	24,977,125	25,280,887	97.5%	98.4%
978	NAT	6	30,246,576	24,546,305	97.5%	98.3%
978	T	6	27,516,248	25,027,456	95.9%	97.8%
151	NAT	8	30,621,478	18,185,435	98.2%	98.3%
151	T	8	29,087,528	28,144,919	97.6%	98.3%
264	NAT	8	24,644,625	26,658,617	95.3%	98.0%
264	T	8	26,136,912	26,776,096	97.3%	98.3%
661	NAT	8	29,339,790	29,590,129	97.9%	98.0%
661	T	8	26,709,899	26,762,749	97.9%	97.4%
692	NAT	8	27,176,676	21,825,289	97.9%	98.1%
692	T	8	29,750,229	22,362,051	97.5%	98.0%
803	NAT	9	28,246,480	24,186,948	96.5%	98.0%
803	T	9	27,429,269	23,233,705	95.2%	98.2%
919	NAT	9	26,005,791	22,981,503	97.7%	97.8%
919	T	9	25,518,861	23,737,734	93.3%	98.3%

Table S2: The following chromosomes and scaffolds from the GRCh38.81 primary assembly were used for RNA-Seq analysis.

**Name**	**Type**	**Name**	**Type**
1	chromosome	GL000213.1	scaffold
2	chromosome	GL000216.2	scaffold
3	chromosome	GL000218.1	scaffold
4	chromosome	GL000219.1	scaffold
5	chromosome	GL000220.1	scaffold
6	chromosome	GL000224.1	scaffold
7	chromosome	GL000225.1	scaffold
8	chromosome	KI270442.1	scaffold
9	chromosome	KI270706.1	scaffold
10	chromosome	KI270707.1	scaffold
11	chromosome	KI270708.1	scaffold
12	chromosome	KI270711.1	scaffold
13	chromosome	KI270713.1	scaffold
14	chromosome	KI270714.1	scaffold
15	chromosome	KI270721.1	scaffold
16	chromosome	KI270722.1	scaffold
17	chromosome	KI270723.1	scaffold
18	chromosome	KI270724.1	scaffold
19	chromosome	KI270726.1	scaffold
20	chromosome	KI270727.1	scaffold
21	chromosome	KI270728.1	scaffold
22	chromosome	KI270731.1	scaffold
MT	chromosome	KI270733.1	scaffold
X	chromosome	KI270734.1	scaffold
Y	chromosome	KI270741.1	scaffold
GL000008.2	scaffold	KI270743.1	scaffold
GL000009.2	scaffold	KI270744.1	scaffold
GL000194.1	scaffold	KI270750.1	scaffold
GL000195.1	scaffold	KI270752.1	scaffold
GL000205.2	scaffold		

Table S3: The primers used for RT-qPCR validation of the six lncRNA candidates. We note that the "C2 – SChLAP1 Exon 3" RT-qPCR assay showed significantly elevated expression of C2 in highly aggressive tumor samples and was used to produce the results reported in Table 3 of the main text. Similarly, the "C2 – SChLAP1 Exons 1-2" RT-qPCR assay showed elevated expression of C2 in aggressive cancers, but high sample-to-sample variability caused this difference to not be called significant (p-value of 0.097 using Welch's t-test with equal variances); this variability may be due to the alternative splicing of exon #2 between patient tissue samples (data not shown).

**Primer[a]**	**Forward**	**Reverse**	**Product size**
C1	AATTTACATCCCCACAAGCAG	GATACCATCTCACACCAGTCAG	126
C2 – SChLAP1 Exons 1-2	GAGTGAGACCAAGAACCCAC	GTGAAAGTGCCTTATACAGGTTG	110
C2 – SChLAP1 Exon 3	GCCTCTGGGGATGCAGATAC	CCGCTCCTCATCATTGGTGA	81
C3	GGAAACCTATGTGAAAGAATGGC	GGAGATATGAGCAACTTTATTAGGATG	98
C4	CGCAAACAGATAAAAGTTAAAGGC	CATCCATAAAGAAATGCTGGCTAG	121
C5	TCTTTCAGGAAGGTTTCCCG	TCTCGATCCCAGTACCTGTAG	130
C6	GTCCTTGCGATAGTTTGCTGAG	ACTATGCAGCCACGAAAAGG	93

[a]Transcript IDs: C1: TCONS_00201747, C2: TCONS_00220343, C3: TCONS_00394362, C4: TCONS_00400757, C5: TCONS_00320203, C6: TCONS_00235780.

Table S4. Transcripts that were found to be significantly different by RNA-Seq differential expression analysis.
The entries with bolded IDs were prioritized for RT-qPCR validation.

Transcript Information					
LncRna Type	Gene Name	Chromosome	Loci	Strand	Transcript ID
novel		19	15592950-15608511	-	**TCONS_00201747**
novel		19	15592950-15608511	-	**TCONS_00201747**
annotated	SCHLAP1	2	180691981-180916981	+	**TCONS_00220343**
novel		2	180916976-180924309	-	**TCONS_00235780**
novel		X	25681633-25687618	+	**TCONS_00394362**
novel		X	25655899-25659825	-	**TCONS_00400757**
annotated	AC009014.3	5	136191471-136192461	+	**TCONS_00320203**
annotated	RP11-442H21.2	10	72274915-72275980	-	TCONS_00048510
annotated	AC009478.1	2	180585992-180601147	+	TCONS_00220341
novel		2	180954678-180959104	-	TCONS_00235788
novel		2	180944147-180946295	-	TCONS_00235785
novel		2	180959229-180963839	-	TCONS_00235789
novel		2	180562646-180564934	-	TCONS_00235770
novel		2	180555550-180561854	-	TCONS_00235769
novel		2	180537527-180554662	-	TCONS_00235768
novel		2	180532884-180537262	-	TCONS_00235767
novel		2	180528816-180532763	-	TCONS_00235766
novel		2	180502736-180506243	-	TCONS_00235760
novel		2	180506868-180508794	-	TCONS_00235761
novel		2	180494804-180499169	-	TCONS_00235758
novel		2	180480887-180485505	-	TCONS_00235753
novel		2	180477777-180480156	-	TCONS_00235752
novel		2	180925734-180926866	-	TCONS_00235781
novel		2	180946478-180949187	-	TCONS_00235786
novel		2	180949313-180954619	-	TCONS_00235787
novel		11	23564245-23565595	-	TCONS_00065519
novel		2	180964664-180966219	-	TCONS_00235790
novel		2	180523410-180524362	-	TCONS_00235764
novel		2	180567461-180569534	+	TCONS_00220340
novel		10	121053929-121067073	-	TCONS_00049559
novel		19	15601765-15603765	-	TCONS_00201749
annotated	CH17-373J23.1	1	145281061-145287043	+	TCONS_00010717
annotated	GS1-114I9.1	7	30416738-30426897	+	TCONS_00344503
novel		10	121054194-121071454	-	TCONS_00051575
annotated	RP11-108P20.2	18	58734695-58758149	-	TCONS_00190630
annotated	C12orf79	12	91991349-92080465	-	TCONS_00094181
novel		X	25734711-25739961	-	TCONS_00400766
novel		8	53399809-53409082	+	TCONS_00365577
annotated	LINC01444	18	14966118-14976705	-	TCONS_00188595
annotated	CTD-2315E11.1	15	90128668-90138242	+	TCONS_00129771
annotated	RP11-1081M5.1	8	53392653-53396625	-	TCONS_00382855
novel		6	110600712-110604572	-	TCONS_00349126
annotated	LINC01444	18	14947514-14979320	-	TCONS_00182635

Table S4. (continued)

		Differential Expression (DE) Analysis			
DE Comparison	DE Tissue	Library Prep	abs(log2(Fold Change))	-LOG(Adj. p-value)	Adj. p-value
Agg vs Ind	Tumor	rRNAd	4.39	3.32	4.80E-04
Agg vs Ind	**Normal**	rRNAd	3.82	2.11	7.74E-03
Agg vs Ind	Tumor	rRNAd	5.36	2.83	1.50E-03
Agg vs Ind	Tumor	rRNAd	4.99	4.86	1.38E-05
Agg vs Ind	**Normal**	rRNAd	4.91	2.49	3.21E-03
Agg vs Ind	**Normal**	rRNAd	5.64	2.10	7.98E-03
Agg vs Ind	Tumor	poly(A)+	3.61	2.06	8.62E-03
Agg vs Ind	Tumor	poly(A)+	7.25	2.10	7.90E-03
Agg vs Ind	Tumor	rRNAd	3.55	3.04	9.08E-04
Agg vs Ind	Tumor	rRNAd	5.04	4.39	4.08E-05
Agg vs Ind	Tumor	rRNAd	4.65	3.22	5.96E-04
Agg vs Ind	Tumor	rRNAd	4.81	4.35	4.42E-05
Agg vs Ind	Tumor	rRNAd	3.69	2.70	2.01E-03
Agg vs Ind	Tumor	rRNAd	3.71	3.09	8.04E-04
Agg vs Ind	Tumor	rRNAd	3.75	3.43	3.70E-04
Agg vs Ind	Tumor	rRNAd	3.96	3.81	1.54E-04
Agg vs Ind	Tumor	rRNAd	3.72	3.34	4.59E-04
Agg vs Ind	Tumor	rRNAd	3.30	3.01	9.77E-04
Agg vs Ind	Tumor	rRNAd	3.84	2.28	5.22E-03
Agg vs Ind	Tumor	rRNAd	4.41	4.43	3.72E-05
Agg vs Ind	Tumor	rRNAd	4.23	3.44	3.63E-04
Agg vs Ind	Tumor	rRNAd	4.61	4.22	5.97E-05
Agg vs Ind	Tumor	rRNAd	4.97	3.43	3.73E-04
Agg vs Ind	Tumor	rRNAd	4.39	3.18	6.64E-04
Agg vs Ind	Tumor	rRNAd	4.82	3.86	1.39E-04
Agg vs Ind	Tumor	rRNAd	4.42	2.03	9.29E-03
Agg vs Ind	Tumor	rRNAd	5.09	2.84	1.43E-03
Agg vs Ind	Tumor	rRNAd	4.06	2.17	6.70E-03
Agg vs Ind	Tumor	rRNAd	3.44	2.41	3.85E-03
Agg vs Ind	Tumor	rRNAd	7.46	3.18	6.57E-04
Agg vs Ind	Tumor	rRNAd	3.82	3.01	9.79E-04
Agg vs Ind	Tumor	poly(A)+	1.74	2.39	4.07E-03
Agg vs Ind	Tumor	rRNAd	1.36	2.53	2.96E-03
Agg vs Ind	Tumor	poly(A)+	6.68	4.30	5.01E-05
Agg vs Ind	Tumor	poly(A)+	1.91	3.27	5.35E-04
Agg vs Ind	Tumor	rRNAd	4.12	4.02	9.51E-05
Agg vs Ind	**Normal**	rRNAd	4.86	2.25	5.61E-03
Agg vs Ind	Tumor	rRNAd	4.90	4.57	2.67E-05
Agg vs Ind	Tumor	poly(A)+	7.35	2.97	1.06E-03
Agg vs Ind	Tumor	poly(A)+	4.73	2.78	1.65E-03
Agg vs Ind	Tumor	poly(A)+	5.31	2.62	2.41E-03
Agg vs Ind	Tumor	poly(A)+	4.21	2.63	2.35E-03
Agg vs Ind	Tumor	rRNAd	6.62	2.97	1.07E-03

Table S4. (continued)

	Average & standard deviation of transcript abundance (TPM) across 6 samples of each subtype							
Max Abundance	Agg **Normal**	+/-	Ind **Normal**	+/-	Agg Tumor	+/-	Ind Tumor	+/-
9.9	10.358	13.139	0.688	0.585	9.905	14.771	0.425	0.188
10.4	10.358	13.139	0.688	0.585	9.905	14.771	0.425	0.188
5.5	0.352	0.385	0.613	0.453	5.510	9.944	0.145	0.117
9.0	0.477	0.399	0.623	0.272	9.013	17.498	0.295	0.137
1.7	1.745	3.009	0.058	0.092	0.417	0.373	0.048	0.029
1.5	1.538	2.671	0.032	0.038	0.340	0.324	0.035	0.059
3.5	0.387	0.296	1.232	0.832	0.328	0.461	3.458	5.219
12.0	4.650	3.891	3.787	6.400	11.968	15.411	0.078	0.192
5.8	0.855	0.429	0.765	0.293	5.838	8.786	0.503	0.235
4.8	0.300	0.238	0.278	0.124	4.773	9.284	0.150	0.096
4.4	0.185	0.105	0.280	0.137	4.382	8.489	0.193	0.137
4.2	0.235	0.190	0.262	0.092	4.183	7.790	0.152	0.087
3.8	0.532	0.167	0.378	0.223	3.770	5.748	0.293	0.130
3.7	0.473	0.298	0.503	0.234	3.663	5.322	0.275	0.105
3.3	0.463	0.275	0.353	0.149	3.287	4.659	0.238	0.074
2.7	0.438	0.390	0.260	0.097	2.695	3.701	0.163	0.074
2.6	0.442	0.274	0.285	0.090	2.620	3.409	0.197	0.098
2.4	0.368	0.274	0.303	0.101	2.425	3.074	0.245	0.062
1.9	0.288	0.267	0.198	0.073	1.932	2.340	0.125	0.104
1.8	0.355	0.298	0.120	0.085	1.830	2.642	0.088	0.024
1.8	0.293	0.223	0.157	0.093	1.807	2.340	0.083	0.081
1.7	0.252	0.172	0.048	0.035	1.660	2.190	0.067	0.044
7.6	0.660	0.493	0.403	0.255	7.643	14.848	0.248	0.183
6.0	0.285	0.248	0.447	0.192	5.955	11.760	0.303	0.185
5.7	0.407	0.315	0.422	0.164	5.658	10.889	0.212	0.134
4.3	0.257	0.288	0.357	0.315	4.323	8.895	0.138	0.115
2.8	0.170	0.182	0.145	0.104	2.827	5.354	0.087	0.063
2.7	0.578	0.302	0.175	0.121	2.703	3.781	0.153	0.136
2.4	0.383	0.273	0.433	0.157	2.397	2.806	0.202	0.161
2.4	0.017	0.024	0.023	0.024	2.377	2.834	0.010	0.013
2.1	0.708	0.525	0.195	0.167	2.093	2.715	0.140	0.087
2.1	0.513	0.113	0.497	0.168	2.053	1.461	0.638	0.185
1.9	0.852	0.257	0.695	0.144	1.898	1.657	0.633	0.151
5.1	0.082	0.070	0.057	0.046	5.147	6.201	0.062	0.057
2.8	1.438	0.676	1.203	0.277	2.772	1.926	0.753	0.321
2.6	0.322	0.126	0.218	0.159	2.647	3.394	0.148	0.200
1.6	1.633	2.753	0.058	0.096	0.328	0.320	0.047	0.049
9.2	0.647	0.332	0.553	0.172	9.167	18.383	0.253	0.168
8.3	0.073	0.070	0.043	0.048	8.260	13.871	0.050	0.048
6.3	1.383	0.918	0.710	0.557	6.252	13.377	0.270	0.231
5.2	0.237	0.197	0.148	0.107	5.158	10.766	0.147	0.152
1.6	0.900	1.238	0.070	0.015	1.592	3.469	0.075	0.063
1.5	0.027	0.033	0.013	0.020	1.532	2.357	0.012	0.012

Table S4. (continued)

Number of samples with transcript abundance above threshold (mean + 3σ) of other classification	
$n_{aggressive}$ where $x_{aggressive} > (mean + 3\sigma)_{indolent}$	$n_{indolent}$ where $x_{indolent} > (mean + 3\sigma)_{aggressive}$
5	0
4	0
4	0
5	0
4	0
4	0
0	3
5	0
5	0
5	0
5	0
5	0
5	0
5	0
5	0
5	0
5	0
5	0
5	0
5	0
5	0
5	0
4	0
4	0
4	0
4	0
4	0
4	0
4	0
4	0
4	0
4	0
4	0
3	0
3	0
3	0
3	0
2	0
2	0
2	0
2	0
2	0
2	0

Table S5. Coordinates and cufflinks attributes for each reconstructed transcript that exhibited significant differences between aggressive and indolent cancers.

Transcript ID	Library Prep	Chromosome	Source	Feature	Start	End	Strand
TCONS_00010717	poly(A)+	1	Cufflinks	exon	145281061	145287043	+
TCONS_00010718	poly(A)+	1	Cufflinks	exon	145281116	145281462	+
TCONS_00048510	poly(A)+	10	Cufflinks	exon	72274915	72275480	-
TCONS_00048510	poly(A)+	10	Cufflinks	exon	72275700	72275980	-
TCONS_00051575	poly(A)+	10	Cufflinks	exon	121054194	121054631	-
TCONS_00051575	poly(A)+	10	Cufflinks	exon	121054747	121055539	-
TCONS_00051575	poly(A)+	10	Cufflinks	exon	121069817	121071454	-
TCONS_00129771	poly(A)+	15	Cufflinks	exon	90128668	90128701	+
TCONS_00129771	poly(A)+	15	Cufflinks	exon	90133397	90133632	+
TCONS_00129771	poly(A)+	15	Cufflinks	exon	90137907	90138242	+
TCONS_00188595	poly(A)+	18	Cufflinks	exon	14966118	14966184	-
TCONS_00188595	poly(A)+	18	Cufflinks	exon	14972351	14976705	-
TCONS_00190630	poly(A)+	18	Cufflinks	exon	58734695	58735329	-
TCONS_00190630	poly(A)+	18	Cufflinks	exon	58741865	58742014	-
TCONS_00190630	poly(A)+	18	Cufflinks	exon	58744338	58744495	-
TCONS_00190630	poly(A)+	18	Cufflinks	exon	58745666	58745791	-
TCONS_00190630	poly(A)+	18	Cufflinks	exon	58747405	58755228	-
TCONS_00190630	poly(A)+	18	Cufflinks	exon	58757473	58758149	-
TCONS_00320203	poly(A)+	5	Cufflinks	exon	136191471	136192461	+
TCONS_00349126	poly(A)+	6	Cufflinks	exon	110600712	110604572	-
TCONS_00382855	poly(A)+	8	Cufflinks	exon	53392653	53395756	-
TCONS_00382855	poly(A)+	8	Cufflinks	exon	53395899	53396625	-
TCONS_00049559	rRNAd	10	Cufflinks	exon	121053929	121054631	-
TCONS_00049559	rRNAd	10	Cufflinks	exon	121054747	121055539	-
TCONS_00049559	rRNAd	10	Cufflinks	exon	121056708	121067073	-
TCONS_00065519	rRNAd	11	Cufflinks	exon	23564245	23565595	-
TCONS_00094181	rRNAd	12	Cufflinks	exon	91991349	91993276	-
TCONS_00094181	rRNAd	12	Cufflinks	exon	91993370	91993529	-
TCONS_00094181	rRNAd	12	Cufflinks	exon	92044395	92080465	-
TCONS_00182635	rRNAd	18	Cufflinks	exon	14947514	14947959	-
TCONS_00182635	rRNAd	18	Cufflinks	exon	14962070	14969572	-
TCONS_00182635	rRNAd	18	Cufflinks	exon	14969676	14969778	-
TCONS_00182635	rRNAd	18	Cufflinks	exon	14969878	14979320	-
TCONS_00182638	rRNAd	18	Cufflinks	exon	14969001	14969572	-
TCONS_00182638	rRNAd	18	Cufflinks	exon	14969676	14969757	-
TCONS_00201747	rRNAd	19	Cufflinks	exon	15592950	15592966	-
TCONS_00201747	rRNAd	19	Cufflinks	exon	15594220	15596956	-
TCONS_00201747	rRNAd	19	Cufflinks	exon	15607569	15608511	-
TCONS_00201749	rRNAd	19	Cufflinks	exon	15601765	15603765	-
TCONS_00220340	rRNAd	2	Cufflinks	exon	180567461	180569534	+
TCONS_00220341	rRNAd	2	Cufflinks	exon	180585992	180601147	+
TCONS_00220343	rRNAd	2	Cufflinks	exon	180691981	180692441	+
TCONS_00220343	rRNAd	2	Cufflinks	exon	180724596	180724690	+
TCONS_00220343	rRNAd	2	Cufflinks	exon	180827241	180827507	+
TCONS_00220343	rRNAd	2	Cufflinks	exon	180916273	180916981	+
TCONS_00235752	rRNAd	2	Cufflinks	exon	180477777	180480156	-
TCONS_00235753	rRNAd	2	Cufflinks	exon	180480887	180485505	-
TCONS_00235758	rRNAd	2	Cufflinks	exon	180494804	180499169	-
TCONS_00235760	rRNAd	2	Cufflinks	exon	180502736	180506243	-
TCONS_00235761	rRNAd	2	Cufflinks	exon	180506868	180508794	-
TCONS_00235764	rRNAd	2	Cufflinks	exon	180523410	180524362	-
TCONS_00235766	rRNAd	2	Cufflinks	exon	180528816	180532763	-
TCONS_00235767	rRNAd	2	Cufflinks	exon	180532884	180537262	-

Table S5. (continued)

Transcript ID	Library Prep	Chromosome	Source	Feature	Start	End	Strand
TCONS_00235768	rRNAd	2	Cufflinks	exon	180537527	180554662	-
TCONS_00235769	rRNAd	2	Cufflinks	exon	180555550	180561854	-
TCONS_00235770	rRNAd	2	Cufflinks	exon	180562646	180564934	-
TCONS_00235780	rRNAd	2	Cufflinks	exon	180916976	180924309	-
TCONS_00235781	rRNAd	2	Cufflinks	exon	180925734	180926866	-
TCONS_00235785	rRNAd	2	Cufflinks	exon	180944147	180946295	-
TCONS_00235786	rRNAd	2	Cufflinks	exon	180946478	180949187	-
TCONS_00235787	rRNAd	2	Cufflinks	exon	180949313	180954619	-
TCONS_00235788	rRNAd	2	Cufflinks	exon	180954678	180959104	-
TCONS_00235789	rRNAd	2	Cufflinks	exon	180959229	180963839	-
TCONS_00235790	rRNAd	2	Cufflinks	exon	180964664	180966219	-
TCONS_00344503	rRNAd	7	Cufflinks	exon	30416738	30426897	+
TCONS_00344505	rRNAd	7	Cufflinks	exon	30424672	30425412	+
TCONS_00365577	rRNAd	8	Cufflinks	exon	53399809	53399837	+
TCONS_00365577	rRNAd	8	Cufflinks	exon	53401480	53401569	+
TCONS_00365577	rRNAd	8	Cufflinks	exon	53405798	53408579	+
TCONS_00365577	rRNAd	8	Cufflinks	exon	53408699	53409082	+
TCONS_00394362	rRNAd	X	Cufflinks	exon	25681633	25687618	+
TCONS_00400757	rRNAd	X	Cufflinks	exon	25655899	25659825	-
TCONS_00400766	rRNAd	X	Cufflinks	exon	25734711	25739961	-

Table S5. (continued)

Transcript ID	Gene ID	Exon #	Class Code	Gene Name	Nearest Reference
TCONS_00010717	XLOC_002031	1	=	CH17-373J23.1	ENST00000618589
TCONS_00010718	XLOC_002031	1	=	CH17-373J23.1	ENST00000618589
TCONS_00048510	XLOC_009939	1	=	RP11-442H21.2	ENST00000491934
TCONS_00048510	XLOC_009939	2	=	RP11-442H21.2	ENST00000491934
TCONS_00051575	XLOC_010578	1	u		
TCONS_00051575	XLOC_010578	2	u		
TCONS_00051575	XLOC_010578	3	u		
TCONS_00129771	XLOC_025043	1	j	CTD-2315E11.1	ENST00000561101
TCONS_00129771	XLOC_025043	2	j	CTD-2315E11.1	ENST00000561101
TCONS_00129771	XLOC_025043	3	j	CTD-2315E11.1	ENST00000561101
TCONS_00188595	XLOC_034484	1	o	LINC01444	ENST00000580867
TCONS_00188595	XLOC_034484	2	o	LINC01444	ENST00000580867
TCONS_00190630	XLOC_034831	1	j	RP11-108P20.2	ENST00000586383
TCONS_00190630	XLOC_034831	2	j	RP11-108P20.2	ENST00000586383
TCONS_00190630	XLOC_034831	3	j	RP11-108P20.2	ENST00000586383
TCONS_00190630	XLOC_034831	4	j	RP11-108P20.2	ENST00000586383
TCONS_00190630	XLOC_034831	5	j	RP11-108P20.2	ENST00000586383
TCONS_00190630	XLOC_034831	6	j	RP11-108P20.2	ENST00000586383
TCONS_00320203	XLOC_059439	1	x	AC009014.3	ENST00000607574
TCONS_00349126	XLOC_065788	1	u		
TCONS_00382855	XLOC_072916	1	=	RP11-1081M5.1	ENST00000521558
TCONS_00382855	XLOC_072916	2	=	RP11-1081M5.1	ENST00000521558
TCONS_00049559	XLOC_012415	1	u		
TCONS_00049559	XLOC_012415	2	u		
TCONS_00049559	XLOC_012415	3	u		
TCONS_00065519	XLOC_016033	1	u		
TCONS_00094181	XLOC_021963	1	j	C12orf79	ENST00000551843
TCONS_00094181	XLOC_021963	2	j	C12orf79	ENST00000551843
TCONS_00094181	XLOC_021963	3	j	C12orf79	ENST00000551843
TCONS_00182635	XLOC_040904	1	j	LINC01444	ENST00000580867
TCONS_00182635	XLOC_040904	2	j	LINC01444	ENST00000580867
TCONS_00182635	XLOC_040904	3	j	LINC01444	ENST00000580867
TCONS_00182635	XLOC_040904	4	j	LINC01444	ENST00000580867
TCONS_00182638	XLOC_040904	1	=	LINC01444	ENST00000581211
TCONS_00182638	XLOC_040904	2	=	LINC01444	ENST00000581211
TCONS_00201747	XLOC_044104	1	u		
TCONS_00201747	XLOC_044104	2	u		
TCONS_00201747	XLOC_044104	3	u		
TCONS_00201749	XLOC_044106	1	u		
TCONS_00220340	XLOC_048106	1	u		
TCONS_00220341	XLOC_048107	1	x	AC009478.1	ENST00000429816
TCONS_00220343	XLOC_048108	1	j	SCHLAP1	ENST00000629145
TCONS_00220343	XLOC_048108	2	j	SCHLAP1	ENST00000629145
TCONS_00220343	XLOC_048108	3	j	SCHLAP1	ENST00000629145
TCONS_00220343	XLOC_048108	4	j	SCHLAP1	ENST00000629145
TCONS_00235752	XLOC_051855	1	u		
TCONS_00235753	XLOC_051856	1	u		
TCONS_00235758	XLOC_051861	1	u		
TCONS_00235760	XLOC_051863	1	u		
TCONS_00235761	XLOC_051864	1	u		
TCONS_00235764	XLOC_051866	1	u		
TCONS_00235766	XLOC_051868	1	u		
TCONS_00235767	XLOC_051869	1	u		

Table S5. (continued)

Transcript ID	Gene ID	Exon #	Class Code	Gene Name	Nearest Reference
TCONS_00235768	XLOC_051870	1	u		
TCONS_00235769	XLOC_051871	1	u		
TCONS_00235770	XLOC_051872	1	u		
TCONS_00235780	XLOC_051877	1	u		
TCONS_00235781	XLOC_051878	1	u		
TCONS_00235785	XLOC_051880	1	u		
TCONS_00235786	XLOC_051881	1	u		
TCONS_00235787	XLOC_051882	1	u		
TCONS_00235788	XLOC_051883	1	u		
TCONS_00235789	XLOC_051884	1	u		
TCONS_00235790	XLOC_051885	1	u		
TCONS_00344503	XLOC_080774	1	=	GS1-114I9.1	ENST00000608195
TCONS_00344505	XLOC_080774	1	=	GS1-114I9.1	ENST00000608195
TCONS_00365577	XLOC_086213	1	u		
TCONS_00365577	XLOC_086213	2	u		
TCONS_00365577	XLOC_086213	3	u		
TCONS_00365577	XLOC_086213	4	u		
TCONS_00394362	XLOC_094849	1	u		
TCONS_00400757	XLOC_096965	1	u		
TCONS_00400766	XLOC_096974	1	u		

Table S6: Differences in expression exhibited by selected RNAs between normal and tumor prostate tissues.

**Candidate**[a]	**Tissue**[b]	**ΔC_T mean ($\pm$ SD)**[c]	***p*-value**[d]
C1	N (n = 78)	8.05 ± 2.14	**0.0005**
	T (n = 67)	6.82 ± 2.02	
C2 – SChLAP1	N (n = 57)	7.85 ± 2.07	**0.024**
	T (n = 62)	6.69 ± 3.26	
C3 – XPLAID	N (n = 56)	12.42 ± 2.84	0.151
	T (n = 62)	11.64 ± 2.97	
C4	N (n = 50)	12.65 ± 2.96	0.172
	T (n = 59)	11.88 ± 2.89	
C5 – AC009014.3	N (n = 51)	9.23 ± 1.90	0.996
	T (n = 53)	9.23 ± 2.48	
C6	N (n = 78)	6.37 ± 1.93	**0.0005**
	T (n = 68)	5.18 ± 2.11	

[a]Transcript IDs: C1: TCONS_00201747, C2: TCONS_00220343, C3: TCONS_00394362, C4: TCONS_00400757, C5: TCONS_004320203, C6: TCONS_00235780. [b]Sample number varied due to sample availability. [c]Lower RT-qPCR thresholds (ΔC_T) represent higher target expression. [d]Using Welch's *t*-test with equal variances.

Table S7: Differences in expression exhibited by selected RNAs between normal and tumor prostate tissues, graded by Gleason score.

**Candidate**[a]	**Grade**[b]	**Tissue**	**ΔC_T mean ($\pm$ SD)**[c]	***p*-value**[d]
C1	Low	N (n = 48)	8.14 ± 2.08	**0.011**
		T (n = 40)	7.00 ± 2.01	
	High	N (n = 30)	7.91 ± 2.27	**0.022**
		T (n = 27)	6.56 ± 2.04	
C2 – SChLAP1	Low	N (n = 36)	8.34 ± 2.12	0.069
		T (n = 38)	7.37 ± 2.36	
	High	N (n = 21)	7.00 ± 1.71	0.155
		T (n = 24)	5.60 ± 4.15	
C3 – XPLAID	Low	N (n = 36)	12.97 ± 2.78	0.242
		T (n = 38)	12.23 ± 2.65	
	High	N (n = 20)	11.41 ± 2.75	0.449
		T (n = 24)	10.71 ± 3.26	
C4	Low	N (n = 29)	13.05 ± 2.86	0.248
		T (n = 35)	12.25 ± 2.59	
	High	N (n = 21)	12.10 ± 3.07	0.422
		T (n = 24)	11.32 ± 3.26	
C5 – AC009014.3	Low	N (n = 27)	9.06 ± 2.02	0.141
		T (n = 31)	8.23 ± 2.20	
	High	N (n = 24)	9.42 ± 1.78	**0.042**
		T (n = 22)	10.65 ± 2.18	
C6	Low	N (n = 48)	6.41 ± 1.91	0.081
		T (n = 41)	5.68 ± 2.02	
	High	N (n = 30)	6.30 ± 1.99	**0.0009**
		T (n = 27)	4.42 ± 2.03	

[a]Transcript IDs: C1: TCONS_00201747, C2: TCONS_00220343, C3: TCONS_00394362, C4: TCONS_00400757, C5: TCONS_00320203, C6: TCONS_00235780. [b]Low grades are Gleason 6 & 7(3+4); high grades are Gleason 7(4+3), 8, 9, & 10. Sample number varied due to sample availability. [c]Lower RT-qPCR thresholds (ΔC_T) represent higher target expression. [d]Using Welch's *t*-test with equal variances.
